# Evidence to support the early introduction of laparoscopic suturing skills into the surgical training curriculum

**DOI:** 10.1186/s12909-020-1986-z

**Published:** 2020-03-06

**Authors:** Benjie Tang, Lin Zhang, Afshin Alijani

**Affiliations:** 1 Surgical Skills Centre, Dundee Institute for Healthcare Simulation, Ninewells Hospital and Medical School, University of Dundee, Dundee, DD1 9SY UK; 2 Department of Surgery, the Second Teaching Hospital of Qingdao University, Qingdao Central Medical Group, Qingdao, China; 3 Ninewells Hospital and Medical School, University of Dundee, Dundee, UK

**Keywords:** Laparoscopic skills training, Evidence-based skills training, Laparoscopic suturing, Surgical training curriculum

## Abstract

**Background:**

The objectives of this study were to investigate the relationship between the acquisition of laparoscopic suturing skills and other operative laparoscopic skills and to provide evidence to determine ideal time and duration to introduce laparoscopic suturing training.

**Methods:**

The first part of the study explored the relationship between the acquisition of laparoscopic suturing skills and proficiency of other operative laparoscopic skills. The second part of the study consisted of an opinion survey from senior and junior trainees on aspects of training in laparoscopic suturing.

**Results:**

One hundred twenty-eight surgical trainees participated in this study. The total scores of task performance of 57 senior surgical trainees in laparoscopic suturing skills consisting of needle manipulation and intracoporeal knot tying were improved significantly after the training course (46.9 ± 5.3 vs 29.5 ± 9.4, *P* < .001), the improvement rate was 59%. No statistically significant correlations were observed between intracorporeal laparoscopic suturing skills and proficiency in the basic laparoscopic manipulative skills assessed before (*r* = 0.193; *P* = 0.149) and after (*r* = 0.024; *P* = 0.857) the training course. 91% of senior trainees and 94% junior trainees expressed that intracorporeal suturing should be introduced at an early stage of the training curriculum.

**Conclusions:**

There was no statistically significant correlation between the performance on basic operative laparoscopic skills (non-suturing skills) and laparoscopic suturing skills observed in this study. The acquisition of basic laparoscopic skills is not a prerequisite for training in intracorporeal suturing and it may be beneficial for the surgical trainees to learn this skill early in the surgical training curriculum. Surgical trainees want to learn and practice laparoscopic suturing earlier than later in their training.

## Background

Although tissue approximation can be achieved by various means, laparoscopic suturing remains indispensable for the execution of surgical operations on the gastrointestinal tract. However, suturing techniques learned in open surgery are not automatically transferred into laparoscopic suturing skills due to the visual constraints and mechanical difficulties and it requires different techniques, skills, and practice to master [1–3]. In a recent survey of Fellowship Council (FC) program directors (PDs), it was demonstrated that up to 56% of entering fellows who had finished their residency surgical training were not able to do laparoscopic suturing [4]. The FC is an association created in 1997 to coordinate general surgery subspecialty fellowships toward the common objective of delivering standardized, quality training through a strong accreditation process [4]. Laparoscopic suturing skill was the area most in need of improvement in a national survey conducted by Nepomnayshy et al. in USA [5]. In the survey, the trainees found laparoscopic suturing skills to be the most deficient skill at the conclusion of residency training, as well as considering them to be the most important to master before completion of fellowship training [5].

Laparoscopic suturing skills are best acquired by training on both inanimate and animate models in the skills laboratories before being attempted in operative clinical practice [6–10]. There is good evidence that suturing skills acquired by simulator training can be translated to operative clinical laparoscopic surgery [11, 12]. Both training and learning of intracorporeal suturing and knot tying can be assessed objectively and this enables assessment of progress in skill acquisition [12–16]. A survey conducted amongst urologists confirmed that hands-on laparoscopy training courses contributed to expansion of laparoscopic practice. In these studies, experience gained from these laparoscopic training courses enabled 61% participants to improve their clinical practice by including intracorporeal suturing in laparoscopic urological operations [17, 18]. In a similar study, Sleiman et al. demonstrated that a short well-guided training course, using the European Academy laparoscopic “Suturing Training and Testing (SUTT) model, significantly improved surgeon’s laparoscopic suturing ability, regardless of their level of experience in laparoscopic surgery [19].

Because of the visual and mechanical constraints, laparoscopic suturing is regarded as a demanding laparoscopic task and is reserved for the more advanced trainees who have mastered the other less taxing laparoscopic component skills in some training curriculums and countries [2, 4, 20]. For instance, the surgical trainees start learning laparoscopic suturing skills formally at year 3 or year 4 of their surgical training curriculum when they need to master this skills for a surgical procedure of laparoscopic fundoplication in general surgery in the United Kingdom [21]. Laparoscopic suturing skills have been an integrated session of the Fundamentals of Laparoscopic Surgery (FLS) curriculum and examination in the USA [22]. Mattar et al. conducted a national survey to assess readiness of general surgery graduate trainees entering accredited surgical subspecialty fellowships in North America. One of the major findings was that 56% of the surgical trainees could not do laparoscopic suturing [4]. Kurashima et al. also showed that only 55% of the teaching hospital had a skills lab and assessment tool to assess the laparoscopic skills including laparoscopic suturing skills of their trainees in Japan [23]. Thus, there has been no objective evidence that it is educationally sound on either early or delayed introduction of laparoscopic suturing skills training despite the significant improvement of surgeon’s laparoscopic suturing skills obtained by attending surgical skills training courses [7, 19]. Hence, there is no available objective data which addresses the issue of the optimal time for the introduction of laparoscopic intracorporeal suturing and knot tying in the surgical curriculum.

In our training centre which is the biggest surgical training centre in the United Kingdom, laparoscopic suturing is restricted to intermediate and advanced laparoscopic skills courses and is usually excluded from the basic ones. The present study was set up to explore this issue by studying the relationship between the acquisition of laparoscopic suturing skills and proficiency in the more basic components of laparoscopic skills to gather objective evidence for early introduction of laparoscopic suturing skills into surgical training program at an early stage. It was also designed to obtain the views of both senior and junior surgical trainees on early versus delayed introduction of laparoscopic intracorporeal suturing.

## Methods

### Overall study design

The study was conducted from April 2016 to September 2017 in the Surgical Skills Centre, Ninewells Hospital and Medical School, University of Dundee, UK. Ninety-two senior and 36 junior surgical trainees were recruited in the study (Table 1). Fifty-seven senior surgical trainees were selected from the 92 by the dates they attended the course to participate the first part of the study. The senior trainees were either specialist registrars year 4–6 with clinical laparoscopic surgical experience in the UK or overseas surgery trainees with equivalent experience. Senior participants were selected from the UK, Europe, Africa, and Asia. Considering the differences of training systems, eligibility of the participants’ level of experience was assessed by the experts at the training centre based on the information provided in their CVs and recommendation letters from the heads of their departments.

**Table 1:** Demographics of participants

Level of experience	Senior surgical trainees (***n*** = 92)	Junior surgical trainees (***n*** = 36)
Age (average)	30 ± 2	27 ± 3
Sex	87% male	64% male
Year of experience in laparoscopic surgery	3.0 ± 2.2	1.0 ± 1.3
Number of cases performed of laparoscopic cholecystectomy	47 ± 8	5 ± 4
Proficiency in laparoscopic suturing	29%	none
Region from	58% from the UK and 42% from overseas	93% from the UK and 7% from overseas

The study consisted of two parts. The first part was designed to investigate the relationship between the acquisition of laparoscopic suturing skills and the proficiency level with other more basic laparoscopic component skills and cognitive knowledge. Six common laparoscopic tasks consisting of port insertion, electrosurgical knife dissection, clipping, scissors cutting and applying an endoloop were selected as the basic operative laparoscopic skills while laparoscopic suturing was considered as a skill at one level up. These tasks were well defined skills for assessment of laparoscopic skills in previous publications [24, 25]. These tasks provided more information on the performance of operative laparoscopic skills than the simple peg transfer etc.

**Fig. 1:**
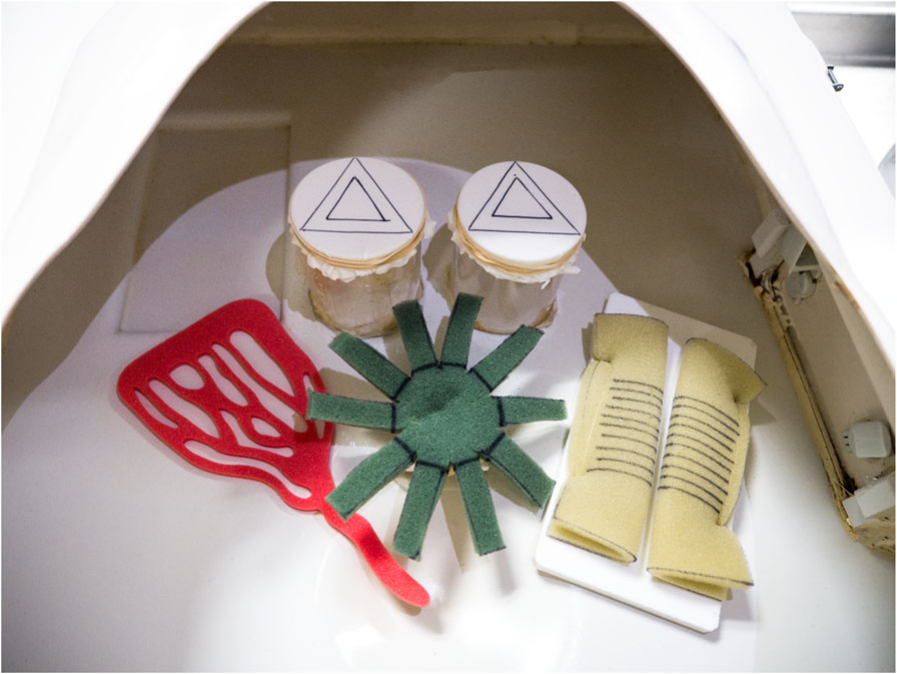
Synthetic models used for basic operative laparoscopic skills (non-suturing skills) and laparoscopic suturing skills (the sponge foam with black marked lines) assessment

**Fig. 2:**
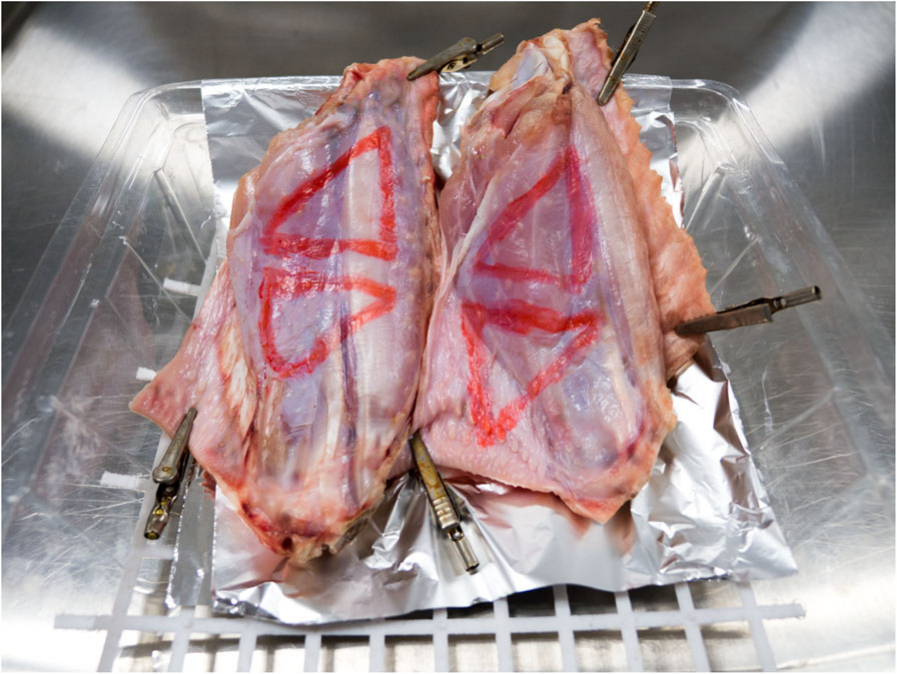
Animal tissue model (turkey wings) marked with triangle for assessment of skills of use electrosurgical hook for dissection

Scissors dissection, clipping, applying an endoloop and laparoscopic suturing were tested on synthetic models (Fig. 1). In the scissors dissection exercise, a double-layered latex membrane was attached with tension to a plastic cylinder using an elastic band. The participant was required to carefully dissect between the black lines and separate the triangular shape from its attachments. Any deviation over the lines or damage to the underlying layer of latex was considered as an error. For the clipping skills test, the participants were asked to select one of the vessels of a synthetic vascular bed and apply two clips, leaving an appropriate distance between the clips to allow safe cutting of the vessel. To accomplish this, the participant must supinate or pronate both wrists to ensure that both jaws of each clip could be seen prior to applying the clip transversely. Participants were also tested on their technique to apply an endoloop onto a simulated appendix. The skills required were to use both hands to position the loop to a marked black line and to tighten it with proper tension. Electrosurgical hook dissection was carried out on turkey wings (Fig. 2). This involved keeping the hook in endoscopic view, and using controlled movement, carrying out dissection in the right tissue plane, with no deviation from the marked lines at the edges of the triangle (accuracy of the dissection) that was drawn on the turkey wing. The laparoscopic suturing task was performed with a 20-cm length with a 3/8 needle (3/0 Polysorb 22 mm taper 3/8 needle, Code GL-303, Medtronic) on a sponge foam with marked lines (Fig. 1).

### Assessors training and reliability in using objective structured clinical examination (OSCE) and observational clinical human reliability analysis (OCHRA) assessment method

The OSCE approach has been in use for the assessment of laparoscopic operative and cognitive skills in the centre since 1990 [26]. This assessment includes: knowledge related to the safe practice of laparoscopic surgery and operative skills. Scores were obtained from each operative task and knowledge station by independent assessor who had adequate training in using this technique. Human Reliability Assessment (HRA) techniques have been in use for several decades in high-risk industries to improve performance and safety. This HRA methodology was modified for use in laparoscopic clinical surgery and was the basis for the validated system of Observational Clinical Human Reliability Assessment (OCHRA) [27].

The assessors had adequate training in using the OSCE and OCHRA techniques. Performance of trainees was assessed during the tests before and after the training courses. Scores were obtained from each operative task and knowledge station by independent assessors. Six experienced consultant surgeons assessed trainees’ performance at the stations. The inter-rater consistency of the OCHRA system assessed was found to be 0.85. The expert panel provided consultation throughout the study and checked the accuracy of the analysis.

It was also the aim to investigate if the proficiency of other basic laparoscopic skills would be a prerequisite for learning the laparoscopic suturing skills by analysing the correlation between the task performance on basic laparoscopic skills and laparoscopic suturing skills. It involved assessment of the proficiency (task performance) of the trainees in six common laparoscopic skills including component steps of laparoscopic intracorporeal suturing.

The ethical committee advised that the consent from the participants was sufficient for ethical approval because of the nature of the study. There were no patients or other conflicting materials involved in this study.

The second part of the study consisted of an opinion survey from 35 senior trainees and 36 junior trainees. These 35 trainees were selected from the 92 trainees who were at the same level of skills as the 57 trainees. The 36 junior trainees were recruited from a basic laparoscopic skills training course. These senior trainees had almost finished their surgical training and had a better understanding of the need for laparoscopic skills training for the surgical trainees [4, 5]. Five expert laparoscopic surgeons who were well recognized and reputed in their field (had experience of more than 200 laparoscopic procedures, taught and trained on a minimum of one laparoscopic course) identified 5 key questions associated with training of laparoscopic suturing skills. They were selected based on aspects of training in laparoscopic suturing: timing, duration and identifying the most technically demanding skills of intracorporeal suturing. Subsequently, these 5 key questions were used to design the questionnaire (Table 2).

**Table 2:** Results of the opinion survey on training in laparoscopic suturing obtained from the senior and junior surgical trainees who participated in the study

	Opinions	Senior trainees (*n* = 35)	Junior surgical trainees (*n* = 36)
Should laparoscopic suturing skills exercise be included in the basic laparoscopic skills training course?	Yes	32 (91%)	34 (94%)
	No	3 (9%)	2 (6%)
What would be ideal duration of the laparoscopic suturing exercise session?	2–4 h	5 (14%)	2 (5%)
	1 half day	3 (9%)	21 (58%)*
	2 half days	20 (57%) *	5 (14%)
	1 day	7 (20%)	8 (22%)
What was (or you consider may be) the most difficult task of laparoscopic suturing?	Needle manipulation	23 (66%)*	8 (22%)
	Bite placement	3 (9%)	2 (2%)
	Knot tying	9 (26%)	28 (78%)*
Is the laparoscopic suturing relevant to your practice?	Agree	33 (94%)	32 (89%)
	Not sure	2 (6%)	3 (9%)
	Disagree	0	1 (3%)
Is the laparoscopic suturing relevant to your current level of experience/ stage in training?	Agree	33 (94%)*	24 (67%)*
	Not sure	2 (6%)	9 (25%)
	Disagree	0	3 (9%)

#### Pre course assessment

This was based on performance of 6 tasks by 57 trainees who attended advanced upper/ lower gastrointestinal laparoscopic surgery training courses. The 6 tasks included the testing of operative laparoscopic skills on electrosurgical hook knife dissection (Fig. 2), clipping, scissor cutting, port insertion, applying an endoloop, and suturing skills (Fig. 1). The nature and purpose of this assessment was explained to all of the participants and formal consents to participate were obtained. The pre-course assessment was conducted in the morning prior to the start of the course. The standard laparoscopic video stack equipment and instruments were used for carrying out all of the tasks in trainer boxes using synthetic and animal tissue models.

Scores of the assessment for the non-suturing laparoscopic operative skills were obtained by use of checklist based on Objective Structured Clinical Examination (OSCE) and Observational Clinical Human Reliability Analysis (OCHRA) [25]. OCHRA and OSCE were used as the more objective means of assessment of laparoscopic operative and cognitive skills in present study. These two assessment methods have been validated previously already [25–27]. The scores for task performance of laparoscopic suturing were assessed using the 29-point checklist method [7]. Both assessment methods had been previously validated [7, 25–28]. Assessments were carried out live by the experienced senior lecturers and consultant surgeons who were experienced in using the assessment methods.

#### Details of the course program

The courses comprised didactic session, live surgery demonstration, anatomy session on a cadaver, expert discussion session and practical hands-on sessions using both synthetic and restructured animal tissue models. The proportion of the time distribution between the didactic and practical sessions was 30 to 70%, emphasizing the predominant hands-on training nature of the course. During the course, the participants undertook an exercise on their ability to overcome the visual constraints of laparoscopic surgery and their efficient equipment and instrument positioning/ manipulation using ergonomic principles to execute dissection and clipping tasks.

Laparoscopic suturing training consisted of skills in ideal ergonomic set up for suturing, handling needle holder, needle manipulation technique, bite placement, and knot tying. The training of laparoscopic suturing skills was at an advanced level, for example, laparoscopic suturing on structures under tension, i.e., repairing hiatal defect during laparoscopic fundoplication. Participants received laparoscopic suturing for two consecutive half days totalling 8 h. Suturing skills included interrupted suturing, continuous suturing, and tumbled square knotting for suturing under tension. Thereafter, the trainees were given the opportunity to apply all of the acquired skills to various surgical procedures on restructured animal tissue models. The simulated procedures consisted of laparoscopic fundoplication, laparoscopic extraction of ductal stones, bowel anastomoses, repair of perforated duodenal ulcer, and gastric bypass etc.

#### Post course assessment

The post-course assessment based on the same six tasks was performed at the end of the course on the 57 participants using the same assessment and scoring systems.

### Opinion survey from trainees

The second part of the study consisted of an opinion survey on aspects of training in laparoscopic suturing: timing, duration, and the most technically demanding skills of the intracorporeal suturing. These 35 senior trainees were selected from the 92 trainees who were at the same level of skills as the 57 who participated the first part of the study. One group of 36 junior trainees were used as a control group. The junior trainees attended a 2-day basic laparoscopic training course. They were in the specialist registrar year 1–2 of their training. The questionnaires were handed in at the end of each course.

### Statistic analysis

Statistical analysis was carried out using the Statistical Package for Social Science version 23 (SPSS, Chicago, Illinois, USA). Data analysis showed that the sample data were not normally distributed. Therefore, the Mann-Whitney test was used to analyse the difference in task performance of all the assessed laparoscopic operative skills before and after the course. Quantitative score data is expressed as mean ± stand deviation (s.d.). The correlations between the performance on the laparoscopic suturing tasks (needle manipulation, bite placement, and intracorporeal knot tying) with other laparoscopic operative skills were analysed by the Pearson’s correlation and linear regression analysis with statistical significance at 0.01.

## Results

### Demographics of participants

Ninety-two senior and 36 junior surgical trainees were recruited in this study (Table 1). Fifty-seven senior trainees participated the first part of the study while 35 senior trainees and 36 junior trainees took part in the second part of the study. The senior trainees were all at a similar level in laparoscopic surgery including laparoscopic suturing skills. They were in years 4–6 of surgical training in the UK and the overseas delegates who had equivalent experience, who were aged between 28 and 32 years. They had 3–4 years experience in laparoscopic surgery, had performed laparoscopic cholecystectomy and laparoscopic appendicectomy independently with numbers varying between 30 to100 cases, averaged around 50 cases. Fifty-eight percent of the participants were from the UK while 42% were from overseas. More than 70% of them were not proficient in laparoscopic suturing. The junior trainees were in year 1–3 of surgical training and were aged between 26 and 30 years. They attended a 2-day basic laparoscopic skills training course.

### Correlation between the basic operative laparoscopic skills and laparoscopic suturing performance

**Fig. 3:**
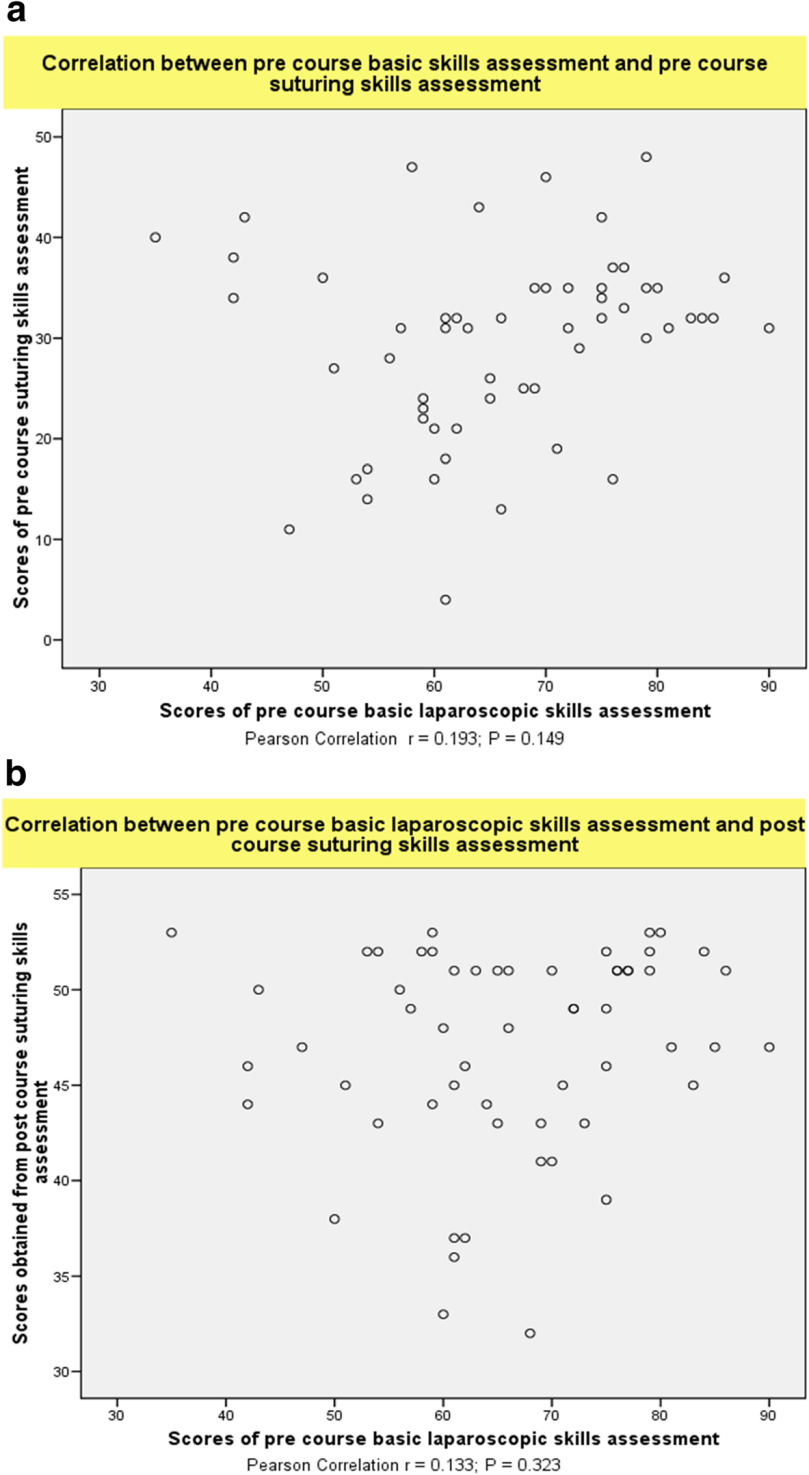
**a** The pre course basic laparoscopic operative skills of participants did not correlate with pre course performance in laparoscopic suturing(*r* = 0.193; *P* = 0.149). **b** The score for the pre course assessment on task performance of other laparoscopic operative skills did not correlate with the task performance of laparoscopic suturing skills assessed post-course(*r* = 0.133; *P* = 0.323)

**Fig. 4:**
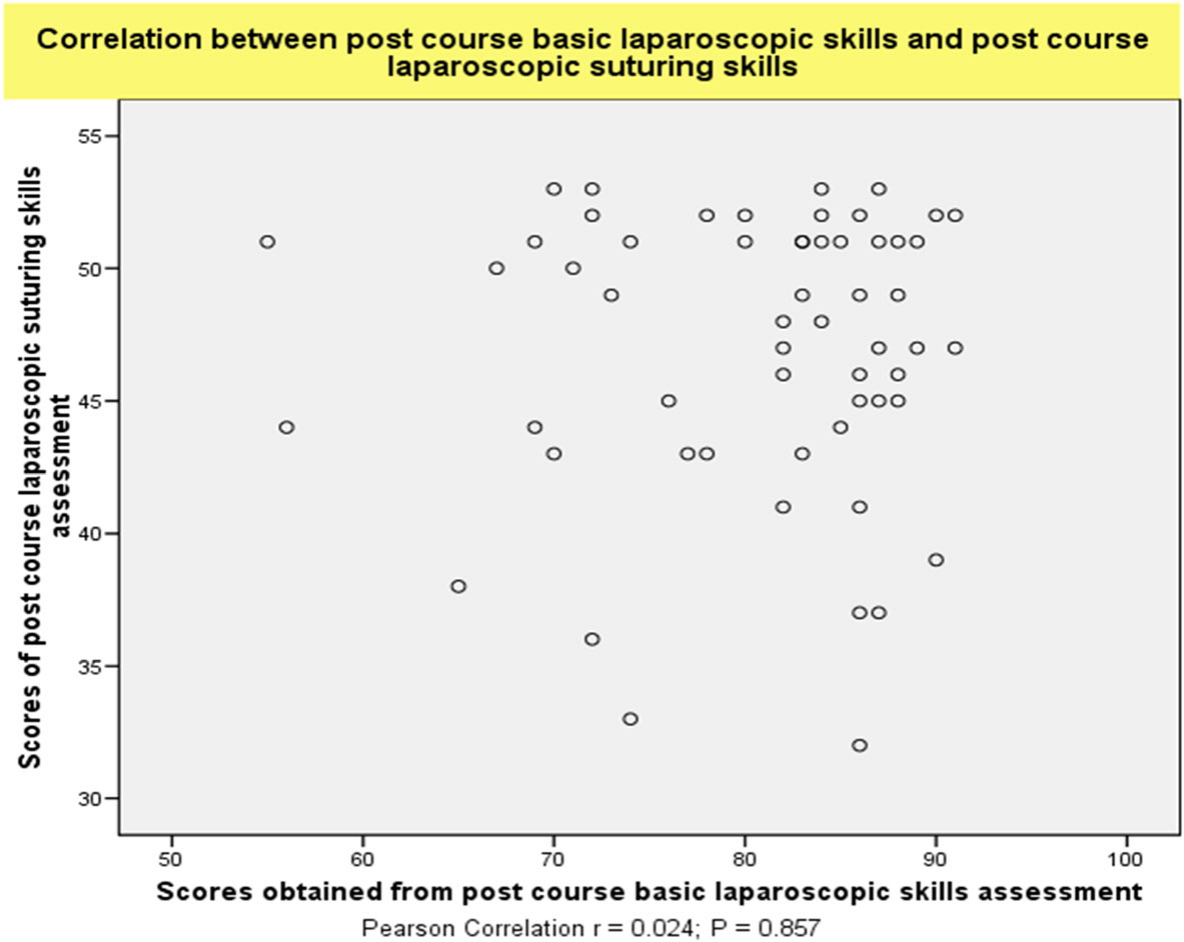
Absence of correlation between the task performances for other basic laparoscopic operative skills and laparoscopic suturing skills during post-course assessment(*r* = 0.024; *P* = 0.857)

The 57 senior trainees were randomly selected by the dates they attended the courses to perform the assessment of basic laparoscopic and suturing tasks before and after completing the advanced training course. In this group, no correlations were observed between the task performance relating to laparoscopic suturing skills and other more basic laparoscopic operative skills (basic operative skills on electrosurgical hook knife dissection, clipping, scissor cutting, port insertion, applying endoloop) before (*r* = 0.193; *P* = 0.149) (Fig. 3a) and after (*r* = 0.133; *P* = 0.323) the course (Fig. 3b). The correlation between the post-course other more basic laparoscopic operative skills (basic operative skills on electrosurgical hook knife dissection, clipping, scissor cutting, port insertion, applying endoloop) and post-course laparoscopic suturing skills was also not significant (*r* = 0.024; *P* = 0.857) (Fig. 4). There was also no correlation between intracorporeal knot tying skills and skills in needle manipulation (*r* = 0.168; *P* = 0.211) and bite placement during (*r* = 0.298; *P* = 0 .024) in the post-course assessment.

### Comparison between the pre and post course task performance (Fig. 5)

**Fig. 5:**
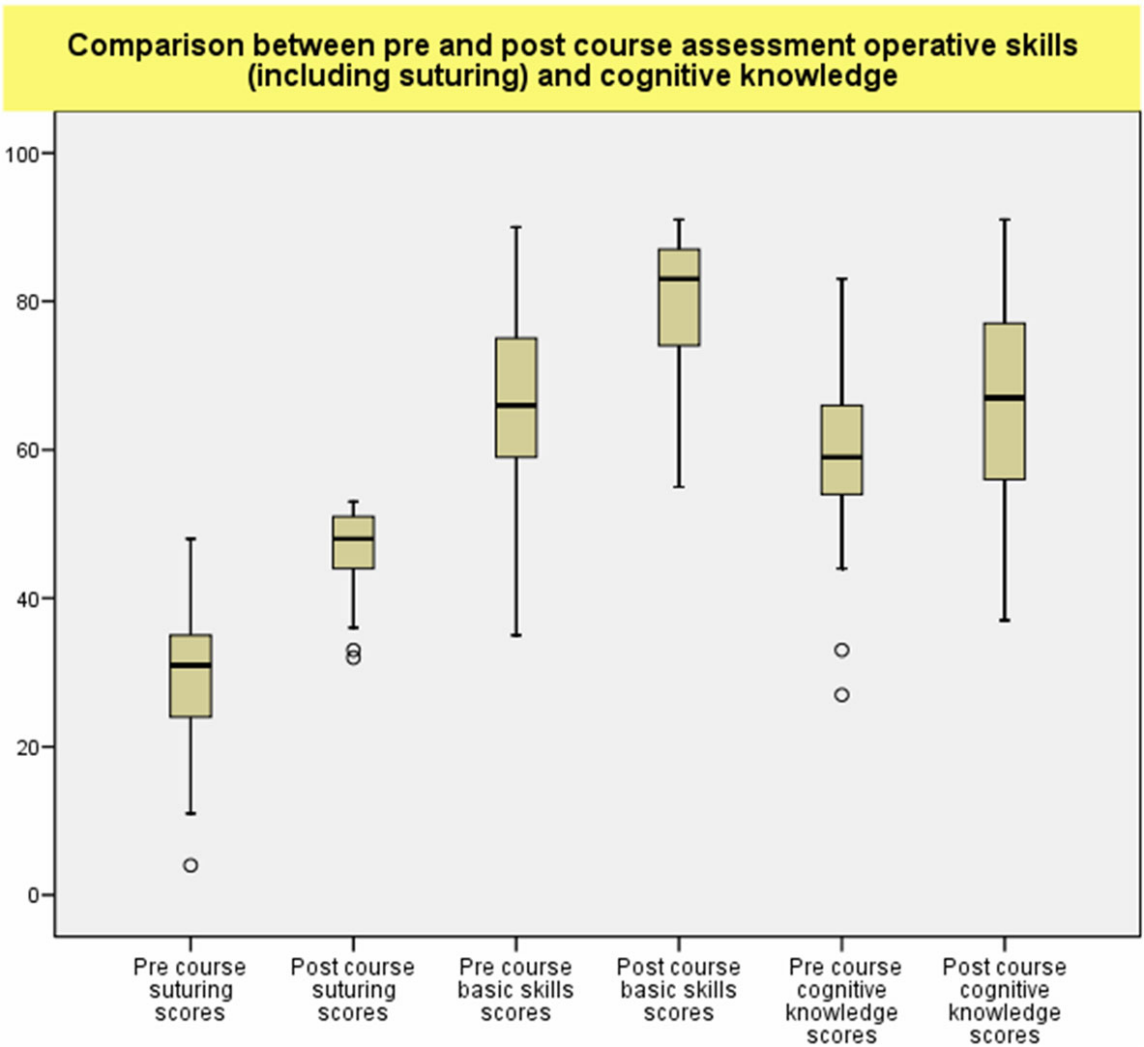
Comparison of task performance in basic operative laparoscopic skills (127.5 ± 10.1 vs 95.5 ± 16.8, *P* < 0.001) and laparoscopic suturing skills (46.9 ± 5.3 vs 29.5 ± 9.4, *P* < .001) before and after the training course. All were improved by the training provided during the course

The total scores of task performance in laparoscopic suturing skills improved significantly after the training course (46.9 ± 5.3 vs 29.5 ± 9.4, *P* < .001), the improvement rate was 59%. The total scores of the other operative laparoscopic skills including port insertion, tissue dissection, using of diathermy hook, clipping, and application of endoloop (127.5 ± 10.1 vs 95.5 ± 16.8, *P* < 0.001) and cognitive knowledge (66.5 ± 16.2 vs 58.8 ± 10.3, *P* < 0.001) also improved significantly in trainees with previous laparoscopic experience, they were improved at a rate of 34 and 14% respectively (Fig. 5).

### Opinions survey on the acquisition of laparoscopic suturing skills

Details of the questionnaires given to the senior (*n* = 35) and junior (*n* = 36) trainees were shown in Table 2. All returned the completed questionnaires (100% compliance). The majority of both groups, 91% of senior trainees and 94% of junior trainees, expressed the view that laparoscopic suturing should be learned at an early stage of their surgical training and should form part of the basic laparoscopic training course. Opinion on the ideal duration of laparoscopic suturing practical sessions differed between the senior trainees who had actually practiced laparoscopic suturing and the junior trainees who had not: 27 (77%) senior trainees opted for two half days (74%) or one full day (26%), whereas 21 (58%) junior trainees considered one half day as being sufficient.

There was a difference between these two groups in their views on the level of difficulty/ complexity of the steps of the intracorporeal suturing task (Table 2). Twenty-three (66%) senior trainees who had practiced the laparoscopic suturing indicated that laparoscopic needle manipulation was the most difficult skill to master, and 9 (26%) considered intracorporeal knot tying was difficult after having attended the training course. In contrast, 28 (78%) junior trainees who had not practiced the laparoscopic suturing predicted that the intra-corporeal knot tying would be the most difficult component of the laparoscopic suturing task.

## Discussion

This is the first study in the surgical literature to investigate the relationship between basic operative laparoscopic skills and laparoscopic suturing skills. The study has demonstrated that there was no statistically significant correlation between the performance on basic operative laparoscopic skills (non-suturing skills) and laparoscopic suturing skills, both before and after attending the laparoscopic training courses. The acquisition of basic operative laparoscopic skills may not be a prerequisite for the acquisition of laparoscopic suturing skills. It has provided scientific basis to explain why training junior operative residents in laparoscopic suturing skills is feasible on a short well-guided training course [7, 19].

Along with the objective evidence, this study has also provided subjective opinions from senior surgical trainees. Surgical trainees indicated their preference for earlier exposure to laparoscopic suturing in their training. The assumption that mastery of basic laparoscopic operative skills is necessary for trainees to benefit from training in laparoscopic suturing is disproved by the findings of the present study.

Skills acquisition from basic to complex skills in laparoscopic and robotic surgery is a profound area to study. The basic operative laparoscopic skills did not correlate significantly to advanced skills such as the laparoscopic suturing skills in our data. This may be similar in acquisition of other complex laparoscopic skills such as operative skills in robotic-assisted surgery. Kowalewski et al. have demonstrated that robotic-assisted surgery required skills distinct from conventional laparoscopy or open surgery [29]. The laparoscopic suturing skills that are involved in several tasks which include handling the needle holders, loading a need onto needle holder, holding a needle at the correct angle and direction, making a bite into the tissue, and finally safely tying a knot. Therefore, laparoscopic suturing skill may be distinct enough that the surgical trainees would benefit from direct experiential training.

Interestingly, we also found that previous experience in laparoscopic suturing did not correlate with the level of laparoscopic suturing performance in the post-course assessment. All of the operative laparoscopic skills including suturing skills were improved significantly by the intensive hands-on training, which was in line with the findings published in the literature [6, 7, 9–11, 13, 19].

To date, skills courses have been designed and developed mainly on the opinions of a panel of expert educators/ tutors without any input from the surgical trainees in some of the surgical training centres. This is perhaps the main reason why laparoscopic suturing is excluded from the basic laparoscopic skills courses [7, 20]. There are, however, other contributing factors which include: (i) few active and well established surgical training centres which run these courses with the necessary in-house expert tutors in laparoscopic suturing, (ii) the implicit belief that laparoscopic suturing may be too difficult for junior trainees and thus counterproductive to their progress if introduced too early in the curriculum, (iii) beside the complexity and difficulty to acquire proficiency in laparoscopic suturing, the other main concern or argument against its earlier introduction in their surgical training is that they will not have the opportunity to apply the skills in their clinical practice, and for this reason, they would deskill very quickly. In the absence of such data, we need to take on board the opinion expressed by the surgical trainees documented by the present study.

In practice, trainees have insufficient access to tutored laparoscopic suturing training sessions on physical models. This is important as the current generations of VR surgical simulators while able to impart the basic component clinical skills, are a long way off providing effective simulation for laparoscopic suturing [8]. The reported study also confirms that laparoscopic suturing skills broaden the clinical applicability of laparoscopy and increases the laparoscopic caseload in both general surgery and urology [16, 17]. For this reason, laparoscopic suturing should be introduced earlier in the surgical curriculum and should certainly be included in basic laparoscopic training courses to prepare them for the opportunity to come [4, 5].

This study also demonstrated that there are different opinions on the ideal duration of laparoscopic suturing exercise sessions between the senior (with experience of laparoscopic suturing) and junior trainees with no previous exposure and who can only guess as to the optimal duration. We consider that one half day to practice suturing skills is not sufficient and recommend two consecutive half days for optimal skill acquisition. This provides adequate exposure for the trainees to practice their laparoscopic suturing to reach proficiency which they can then translate to their practice in the operating room [12, 24, 28].

The survey showed a significant difference in the identification of the difficult steps of laparoscopic suturing. The majority (66%) of the senior trainees indicated that laparoscopic needle manipulation was the most difficult component step, whereas the majority (78%) of junior trainees predicted that the intra-corporeal knot tying would be the most difficult component of the suturing task. The view expressed by junior trainees should not be overlooked as it indicates the need for a precise clear description of the sequential component steps of the intracorporeal knot tying to junior trainees. The senior trainees had obviously advanced beyond this perceived difficulty with knot tying and thus did not identify it as a particularly difficult problem.

Despite the evidence to support the feasibility and efficiency of early introduction of laparoscopic suturing skills into the surgical training curriculum, studies have shown that there was a modest decrement in performance of laparoscopic suturing skills after 6 months of training [30–32]. Therefore, it is fundamentally important to understand that the surgical trainees may become deskilled if their laparoscopic suturing skills are not used in the operating room or maintained with repeated practice in a simulated setting [30]. Mashaud et al. and Scerbo et al. demonstrated that an ongoing structured training programme helped to maintain proficiency of laparoscopic suturing skills [31, 32]. Therefore, a retention interval and refresher session should be provided for the junior trainees who do not have adequate exposure in the operating room to reinforce and maintain laparoscopic suturing skills [9, 15, 30, 32]. The portable laparoscopic simulator and virtual reality simulator have been proven to be valid and effective for this purpose [12, 15, 24].

## Limitations

This study was not conducted in a randomized controlled trial. However, we were aware that this was not achievable during the time when this study was conducted, as we did not have control of choosing the candidates though we assessed their eligibility to attend the course. The study was also not designed to compare the outcome of an early versus late introduction of laparoscopic suturing skills in the surgical training curriculum, thus, there was no data to demonstrate whether laparoscopic suturing skill benefits from prior training in basic operative laparoscopic skills (non-suturing skills) or not. There was no assessment on the actual performance of the senior trainees in the operating room after the course because the study was not designed to assess the transferability of laparoscopic suturing skills from a skills lab into operating room, as many studies have demonstrated this already [10–14, 17, 18, 25].

Senior surgical trainees considered that needle manipulation was the most difficult component for laparoscopic suturing skills whereas the junior group thought the knot tying was the difficult task. We did not conduct a study to investigate this different opinion further. This may be of importance for laparoscopic suturing skills training when teaching surgical trainees at different levels of experience and allocating time on each task during the exercise. This will be an area for future study. We were also fully aware that this study was mainly based on our own institutional data to provide evidence to support the early introduction of laparoscopic suturing skills into surgical training curriculum. Thus, adaption of this practice should be tailored to meet the requirement of the systems that would be beneficial for the surgical trainees to improve their surgical performance.

## Conclusions

There was no statistically significant correlation between the performance on basic operative laparoscopic skills (non-suturing skills) and laparoscopic suturing skills observed in this study. The acquisition of basic laparoscopic skills is not a prerequisite for training in intracorporeal suturing and it may be beneficial for the surgical trainees to learn this skill early in the surgical training curriculum. Surgery trainees want to learn and practice laparoscopic suturing earlier rather than later in their training.

## Data Availability

The datasets produced and analysed in this study are available from the corresponding author on request.

## Abbreviations

CVs: Curriculum Vitaes
FC: Fellowship Council
FLS: Fundamentals of Laparoscopic Surgery
OCHRA: Observational Clinical Human Reliability Analysis
OSCE: Objective Structured Clinical Examination
PDs: Program Directors
SUTT: Suturing Training and Testing

## References

[1] Cuschieri A (1995). Whither minimal access surgery: tribulations and expectations. Am J Surg.

[2] Romero P, Brands O, Nickel F, Muller B, Gunther P, Holland-Cunz S (2014). Intracorporal suturing – driving license necessary?. J Pediatr Surg.

[3] Watanabe Y, McKendy KM, Bilgic E, Enani G, Madani A, Munshi A, Feldman LS, Fried GM, Vassiliou MC (2016). New models for advanced laparoscopic suturing: taking it to the next level. Surg Endosc.

[4] Mattar SG, Alseidi AA, Jones DB, Jeyarajah DR, Swanstrom LL, Aye RW, Wexner SD, Martinez JM, Ross SB, Awad MM, Franklin ME, Arregui ME, Schirmer BD, Minter RM (2013). General surgery residency inadequately prepares trainees for fellowship: results of a survey of fellowship program directors. Ann Surg.

[5] Nepomnayshy D, Alseidi AA, Fitzgibbons SC, Stefanidis D (2016). Identifying the need for and content of an advanced laparoscopic skills curriculum: results of a national survey. Am J Surg.

[6] Soper NJ, Hunter JG (1992). Suturing and knot tying in laparoscopy. Surg Clin North Am.

[7] Aggarwal R, Hance J, Undre S, Ratnasothy J, Moorthy K, Chang A, Darzi A (2006). Training junior operative residents in laparoscopic suturing skills is feasible and efficacious. Surgery.

[8] Botden SM, Torab F, Buzink SN, Jakimowicz JJ (2008). The importance of haptic feedback in laparoscopic suturing training and the additive value of virtual reality simulation. Surg Endosc.

[9] Stefanidis D, Korndorffer JR, Markley S, Sierra R, Scott DJ (2006). Proficiency maintenance: impact of ongoing simulator training on laparoscopic skills retention. J Am Coll Surg.

[10] Stefanidis D, Sierra R, Korndorffer JR, Dunne JB, Markley S, Touchard CL, Scott DJ (2006). Intensive continuing medical education course training on simulators results in proficiency for laparoscopic suturing. Am J Surg.

[11] Korndorffer JR, Dunne JB, Sierra R, Stefanidis D, Touchard CL, Scott DJ (2005). Simulator training for laparoscopic suturing using performance goals translates to the operating room. J Am Coll Surg.

[12] Fried GM, Feldman LS, Vassiliou MC, Fraser SA, Stanbridge D, Ghitulescu G, Andrew CG (2004). Proving the value of simulation in laparoscopic surgery. Ann Surg.

[13] Dubrowski A, Park J, Moulton CA, Larmer J, MacRae H (2007). A comparison of single- and multiple-stage approaches to teaching laparoscopic suturing. Am J Surg.

[14] Van Sickle KR, Baghai M, Huang IP, Goldenberg A, Smith CD, Ritter EM (2008). Construct validity of an objective assessment method for laparoscopic intracorporeal suturing and knot tying. Am J Surg.

[15] Pearson AM, Gallagher AG, Rosser JC, Satava RM (2002). Evaluation of structured and quantitative training methods for teaching intracorporeal knot tying. Surg Endosc.

[16] Dubrowski A, Larmer JC, Leming JK, Brydges R, Carnahan H, Park J (2006). Quantification of process measures in laparoscopic suturing. Surg Endosc.

[17] Allen JW, Rivas H, Cocchione RN, Ferzli GS (2003). Intracorporeal suturing and knot tying broadens the clinical applicability of laparoscopy. JSLS..

[18] Pareek G, Hedican SP, Bishoff JT, Shichman SJ, Wolf JS, Nakada SY (2008). Skills-based laparoscopy training demonstrates long-term transfer of clinical laparoscopic practice: additional follow-up. Urology.

[19] Sleiman Z, Tanos Y, Van Belle Y, Carvalho JL, Campo R (2015). The European academy laparoscopic “suturing training and testing (SUTT)” significantly improves surgeons’ performance. Facts Views Vis Obgyn.

[20] Wade TJ, Lorbeer K, Awad MM, Woodhouse J, DeClue A, Brunt LM (2015). Outcomes of a proficiency-based skills curriculum at the beginning of the fourth year for senior medical students entering surgery. Surgery..

[21] https://www.iscp.ac.uk/static/public/syllabus/syllabus_gs_2016.pdf. Accessed 18 Oct 2019.

[22] Sroka G, Feldman LS, Vassiliou MC, Kaneva PA, Fayez R, Fried GM (2010). Fundamentals of laparoscopic surgery simulator training to proficiency improves laparoscopic performance in the operating room- randomized controlled trial. Am J Surg.

[23] Kurashima Y, Watanabe Y, Ebihara Y, Murakami S, Shichinohe T, Hirano S (2016). Where do we start? The first survey of surgical residency education in Japan. Am J Surg.

[24] Johnston TJ, Tang B, Alijani A, Tait I, Steele RJ, Ker J (2013). Nabi G; surgical simulation group at the University of Dundee. Laparoscopic surgical skills are significantly improved by the use of a portable laparoscopic simulator: results of a randomized controlled trial. World J Surg.

[25] Tang B, Hanna GB, Carter F, Adamson GD, Martindale JP, Cuschieri A (2006). Competence assessment of laparoscopic operative and cognitive skills: objective structured clinical examination (OSCE) or observational clinical human reliability assessment (OCHRA). World J Surg.

[26] Cohen R, Reznick RK, Taylor BR, Provan J, Rothman A (1990). Reliability and validity of the objective structured clinical examination in assessing surgical residents. Am J Surg.

[27] Tang B, Hanna GB, Joice P, Cuschieri A (2004). Identification and categorization of technical errors by Observational Clinical Human Reliability Assessment (OCHRA) during laparoscopic cholecystectomy. Arch Surg.

[28] Moorthy K, Munz Y, Dosis A, Bello F, Chang A, Darzi A (2004). Bimodal assessment of laparoscopic suturing skills: construct and concurrent validity. Surg Endosc.

[29] Kowalewski KF, Schmidt MW, Proctor T, Pohl M, Wennberg E, Karadza E, Romero P, Kenngott HG, Müller-Stich BP, Nickel F (2018). Skills in minimally invasive and open surgery show limited transferability to robotic surgery: results from a prospective study. Surg Endosc.

[30] Castellvi AO, Hollett LA, Minhajuddin A, Hogg DC, Tesfay ST, Scott DJ (2009). Maintaining proficiency after fundamentals of laparoscopic surgery training: a 1-year analysis of skill retention for surgery residents. Surgery..

[31] Mashaud LB, Castellvi AO, Hollett LA, Hogg DC, Tesfay ST, Scott DJ (2010). Two-year skill retention and certification exam performance after fundamentals of laparoscopic skills training and proficiency maintenance. Surgery..

[32] Scerbo MW, Britt RC, Montano M, Kennedy RA, Prytz E, Stefanidis D (2017). Effects of a retention interval and refresher session on intracorporeal suturing and knot tying skill and mental workload. Surgery..

